# INFLUENCE OF PERSON AND ENVIRONMENTAL FACTORS ON OLDER ADULT ENGAGEMENT IN VIDEO TELEHEALTH

**DOI:** 10.1093/geroni/igad104.0479

**Published:** 2023-12-21

**Authors:** Megan Gately, Dylan Waller, Matthew Maynard, Lauren Moo

**Affiliations:** VA Bedford Health Care System, Bedford, Massachusetts, United States; Center to Improve Veteran Involvement in Care (CIVIC), Portland, Oregon, United States; Veterans Health Administration, Forest City, North Carolina, United States; Veterans Health Administration, Bedford, Massachusetts, United States

## Abstract

Rural patients at Veterans Health Administration (VHA) are older and have more complex chronic conditions than Veterans receiving care outside VHA. Rural patients face challenges accessing care, particularly specialty services which are often in urban areas. Though video telehealth can increase access to care by older patients, video was underutilized by this group even during the rapid increase in use of video in response to COVID. The unequal uptake of video telehealth by older patients during a time when there were limited options suggests a gap in our understanding of the factors involved with older adults’ utilization of video telehealth. To explore these factors, we interviewed occupational therapy practitioners who were high users of video telehealth (N=27) about their experiences with video telehealth. According to interviews, successful utilization of video telehealth involves consideration of complex factors operating at the person (i.e., patient and clinician) and environmental levels. Person-level factors for the patient and/or family caregiver include technological literacy, sensory impairments, and stress tolerance. Clinician person-level factors include confidence with technology, attitudes towards video (including perceived benefits for clinical care), and specific skills like communication and flexibility. Environmental level factors include the physical setting (e.g., whether the patient/clinician are in a private space), availability of technical assistance for patients and clinicians, caregiver assistance on the patient side, and presence of a device and adequate broadband for patients. Elucidating the network of factors involved with video telehealth may enable sustained integration of video telehealth by rural patients most in need of care.

